# Impact of food price inflation on stunting in under five aged children in Bangladesh

**DOI:** 10.1186/s13561-024-00549-9

**Published:** 2024-08-29

**Authors:** Sheikh Sifat Sadikeen, Nazmul Haque, Md Miraj Hossain, Md Jamal Uddin

**Affiliations:** 1 Department of Statistics, Shahjalal University of Science and Technology, Sylhet, 3114 Bangladesh; 2 Faculty of Graduate Studies, Daffodil International University, Savar, Dhaka, 1216 Bangladesh

**Keywords:** Inflation, Malnutrition, Stunting, Food Price, BDHS, Under five aged, Children, Bangladesh

## Abstract

**Background:**

Currently, food price inflation is a widespread issue in Bangladesh as well as the rest of the world. Malnutrition is a common issue among children that can have long-lasting effects on their development and overall health. There have been lots of studies conducted to identify the factors responsible for child malnutrition, but inflation is rarely considered a factor in child malnutrition. We aimed to determine the relationship between food price inflation and stunting (Height-for-Age Z-score (HAZ)) in children under five years of age in Bangladesh.

**Method:**

The study utilized food price data from the World Food Programme database and malnutrition (stunting) information from the 2014 and 2017-18 Bangladesh Demographic Health Surveys (BDHS). This includes the total study period from 2009 to 2018. Food prices were linked to the BDHS dataset using each child’s birth month. For each child, the average food prices from 9 months prior to 5 months post-birth, including their birth month, were recorded to calculate month-to-month inflation. This inflation was computed for rice (coarse), oil, wheat flour, and lentils by comparing the price sum of each item from one month to the previous month and dividing by the total price of the preceding month. A generalized linear regression model was used to assess the relationship between food price inflation and stunting, with stunting as the dependent variable. Other explanatory variables included wealth index, sex of the child, height, weight, mother’s education, respondent’s current pregnancy, and breastfeeding status.

**Results:**

Our study has revealed that food price inflation has a significant negative effect on stunting, with a coefficient of -0.127 (*p* < 0.001). Furthermore, we have identified several other factors that have also significantly negative associations with stunting, including the wealth index (*p* < 0.001), mother’s education level (*p* < 0.001), mother’s pregnancy status (*p* < 0.001), breastfeeding (*p* < 0.001), child’s age (*p* < 0.001). child’s weight (*p* < 0.001) has significantly positive effect on stunting. However, we did not find any significant differences in stunting between boys and girls.

**Conclusion:**

In conclusion, the findings of this study underscore the significant negative impact of food price inflation on child stunting, emphasizing the need to acknowledge this factor alongside others. These results highlight the critical role of addressing food price inflation as a key determinant of stunting, in conjunction with various other contributing factors, in efforts to combat childhood malnutrition.

**Supplementary Information:**

The online version contains supplementary material available at 10.1186/s13561-024-00549-9.

## Introduction

Malnutrition, in overall, adding undernutrition (wasting, stunting, underweight), inadequate vitamins or minerals, overweight, obesity, and resulting diet-related noncommunicable diseases. The World Health Organization (WHO) defines malnutrition as a condition brought on by deficiencies, overconsumption, or unbalanced nutrient intake [1].

Malnutrition refers to two major types of conditions. One of them is “undernutrition,” which includes underweight (weighing less than other children of the same age), stunting (being too short for one’s age group), wasting (weighing less than one’s height), and micronutrient deficiency (lack of essential vitamins and minerals). Both undernutrition and overnutrition are considered forms of malnutrition. Insufficient intake of protein and calories, as well as a deficiency in micronutrients such as vital vitamins and minerals, are all aspects of undernutrition [2]. Child malnutrition is a major global crisis, with 148.1 million stunted children and 45 million wasted children according to the year of 2022 [3]. In 2014 about 158.6 million children under-fives were stunted worldwide [4], and about 155 million children under-fives were stunted and 52 million were wasted worldwide in 2017 [5]. WHO also estimates that 5.4 million under-five aged children die annually (2017 report) [6]. According to statistics from around the world, in 2012, stunting, underweight, and wasting affect 7.3%, 13.4%, and 21.9% of children under the age of five, respectively [7]. After 2000, improving children’s nutrition has become a global priority [4, 8].

Children who are stunted or wasted suffer both short- and long-term consequences of different health issues such as poor motor development, obesity, morbidities [9]. The nutritional condition of children is a crucial indicator of poverty in a society, and there is a connection between poverty, malnutrition, and disease. A number of causes can lead to malnutrition in children, but they typically revolve around bad/low food quality, severe and recurrent viral infections, inadequate food intake, or frequently a combination of the three [10]. These circumstances directly affect a population’s ability to meet needs such as obtaining food, healthcare and shelter, as well as their general standard of living [11]. As a result, assessing children’s nutritional status not only measures their health and survival, but it also gives an indirect measure of the overall population’s quality of life.

The World Food Program (WFP) referred to the increases in food prices that occurred in 2007 and 2008 as the “silent tsunami,” which were brought on by a variety of connected and interrelated variables. In actuality, the cost of basics began to rise a few years before 2008, when it peaked. It is crucial to look at the additional causes raising commodity costs in addition to the fact that Bangladesh has a smaller scale economy dependent on agro-based productions [12].

From the time before its independence until now, Bangladesh has been heavily reliant on rice grain, and after achieving its freedom, need has grown. A war-torn nation takes a very long time to recover. As a result, towards the end of 1980, inflation was a concern. After that time, however, it is driven by both the rising cost of fuel and the worldwide price increase of the global commodity. Overall, there was an increase in food prices. As a result, such price fluctuations are detrimental to people’s standard of life or health. Also, that would hinder human progress in the future, particularly for the impoverished [13].

Moreover three-fifths of the population of Bangladesh, a country with a high density of people, lives below the poverty line, making it one of the world’s poorest developing nations. Due to overpopulation, unemployment, poverty, and limited access to proper food and healthcare services, infectious diseases and malnutrition are pervasive in this community [14]. It has been found that poor food and malnutrition are closely associated with higher morbidity in children living in poverty. Protein energy malnutrition (PEM) impairs development, raises morbidity and death rates, and stunts psychological and intellectual growth [15].

In underdeveloped countries, malnutrition is the cause of approximately 2.3 million child deaths each year, or around 41% of all child deaths in this age group [16]. Bangladesh is a developing agricultural nation where over 80% of the population relies on agriculture in some capacity, with 48% directly involved in farming. Bangladesh was identified by the Food and Agricultural Organization (FAO) as one of the 37 countries going through a “crisis” because of the rise in food prices. In fact, rising food prices have become a significant issue for citizens of the community [17].

Malnutrition is not an easy problem with a single solution. Malnutrition is caused by several interconnected and hierarchically interconnected determinants [18]. Food insecurity, poor maternity and childcare, access to essential health services, such as safe water supply, as well as harmful living conditions such as open defecation, are among the most important direct determinants of health [19, 20]. These underlying causes are then modified by capacity, resources, environmental conditions, governance, national and global contexts, as well as economic, political, and social factors [21].

The objectives of this study are to examine the levels and trends of stunting (Height-for-Age Z-score (HAZ)) among children under five years of age in Bangladesh and to assess their overall nutritional health by comparing food price data. This information will aid in evaluating current child development policies and in formulating impactful new strategies to improve the health of these children. Specifically, this study focuses on two main factors: inflation and malnutrition, with the latter being defined solely as stunting (HAZ) in children.

## Literature review

There are various studies [22–28] in the literature that investigated the influence of inflation on child nutritional outcome. In terms of costs, a study has been done to determine how variations in rice prices affect child underweight in Bangladesh using annual data from 1992 to 2002. The study showed that there was a strong correlation between rice prices and underweight (corr. coef. = 0.91, *p* = 0.001) using aggregate data [28]. Another study utilized panel data from 1993 to 1997 to examine how the drought in Zimbabwe affected children’s growth [27]. One study that used annual data discovered that the reduction in Russia’s economic output between 1996 and 1998 impacted six nutritional health indices [25]. Between 1995 and 1996, the impacts of food aid and a shock (drought) on child growth in Ethiopia were identified [24]. There was a significant pro-cyclical association between Colombian child mortality and price changes for Arabica coffee, even when a counter-cyclical relationship might be anticipated. This study, focusing on medium- to long-term effects, was also based on annual data [22]. Moreover, multiple studies have been conducted to replicate the effects of crises on the economic conditions of countries, subnational territories, and families during the global food and fuel price crisis. For instance, a study was conducted on the impact of the food price issue in nine impoverished South Asian, African, and Latin American countries. It was found that whether a household was a net buyer or net seller was the primary driver of diverse impacts. Overall, the findings indicate that the nine countries under investigation experienced an increase in the number of impoverished individuals and the depth of poverty, as the effects on net buyers outweighed those on net sellers [23]. The researchers used a poverty module attached to an economy-wide GE simulation model to study Mozambique specifically, finding negative impacts on poverty. The rise in poverty, especially in urban areas, was primarily due to the increasing cost of fuel, though it was also influenced by rising food prices [26].

There have been a few international [22–27] studies on the impact of inflation on child malnutrition. However, this topic is rarely researched in Bangladesh. And recently, price inflation is expanding at an alarming rate, complicating people’s lives. Children’s health may suffer as a result of rising inflation. That is why this study is being undertaken to determine whether inflation has a substantial impact on child health in Bangladesh.

A cross-sectional approach is applied in this study to find the relationship between food price inflation and stunting of under five aged children. Figure 1 shows the overall steps used to perform the full analysis.

## Methodology

**Fig. 1:**
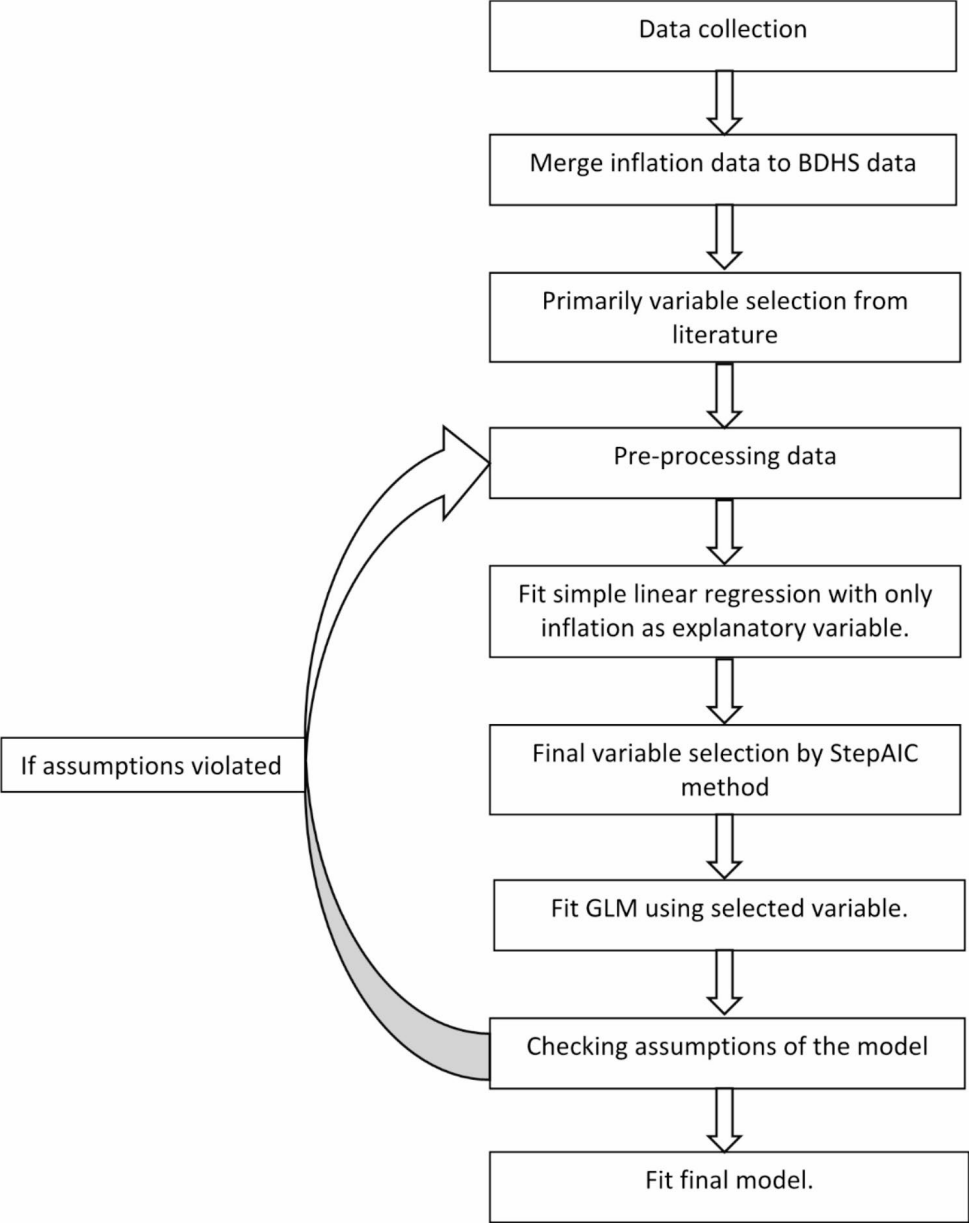
A flow chart of methodology

### Data sources

A cross sectional study about the effect of inflation on stunting among children under the age of five is being carried out using the secondary data from 2014 and 2017–18 Bangladesh Demographic Health Survey [29](BDHS) datasets, which contain the data about the nutritional status of children and their associated factors such as child characteristics, mother characteristics, household characteristics, etc. [30].

BDHS conducted their seventh survey in 2014 and their eighth in 2017–18 which are the most recent dataset so far. The 2014 DHS collected data from 17,989 households across seven divisions of Bangladesh, of which 17,300 completely interviewed. Meanwhile, in the 2017-18 DHS, data from approximately 20,250 randomly selected households from eight divisions was collected. An important note is that Mymensingh is included in the 2017–18 dataset as a division, but in 2014 it was not.

For the lack of information about food inflation in BDHS data, the food price data is collected from the WFP Price Database. It covers items such as maize, rice, beans, seafood, and sugar for 76 nations and 1,500 markets. In the context of Bangladesh, the database contains the data about the price of several varieties of rice such as coarse, BR-8/ 11/, Guti Sharna, medium grain, oil, wheat flour, lentils (masur), etc., from 1998 to 2022, for every division and its surrounding areas [29].

### Data management

The data is first filtered by the mother of the children who is a permanent resident of the household, reducing the dataset to 14,958 under-five-year-olds. Children under the age of six months were excluded from the dataset because inflation was evaluated from nine months before birth to six months after birth. The prior nine months of the child’s birth, as well as the six months following, are calculated into the dataset from the child’s CMC birth month for this reason. CMC date is defined as the number of months since 1900: cmc=(year − 1900)∗12 + month [30]. In this study, inflation refers to month-to-month inflation of the food commodities (rice, oil, lentils, and wheat). Only six divisions—Dhaka, Chittagong, Barisal, Rajshahi, Khulna, and Sylhet—are considered because the pricing dataset for food commodities does not include prices for the Rangpur division, and the 2014 BDHS dataset does not include individual data for the Mymensingh division. Finally, inflation data has been combined with the BDHS dataset for the nine months preceding the child’s birth and the six months after the child’s birth and its corresponding district. It is important to note that the mean for a continuous variable and the mode for a categorical variable are used to impute missing values.

### Variable selection

#### Response variable

In this study, malnutrition (stunting) was considered as the response variable. HAZ (height for age) was used as a continuous variable, for which information is available in the dataset.

### Explanatory variables

#### Main interested variable

Food Inflation (average of 15 months).

#### Child characteristics

Age of child, Weight of child, Gender.

#### Mother characteristics

Mother education Level, Respondent’s current pregnancy status, Whether the respondent is breastfeeding their child right now, Respondent’s occupation as collected in the country, most recent partner or husband’s highest education level, Age of the respondent’s husband or partner, Religion, Total number of children ever born.

#### Household characteristics

Wealth Index, Toilet facilities shared with other households, Total number of living children, Sex of the head of the household, Type of place of residence, Primary source of drinkable water by household members, Toilet facility type in the household, Type of cooking fuel.

### Data analysis

Because of the scarcity of data in all areas of interest, only rice (coarse), oil, wheat flour, and lentils are used to calculate inflation in this study. The following equation provides the basis for calculating food price inflation in or study [31]:

$$\:{{\pi}}_{\text{k}\left(\text{m}\right)\text{i}}=\frac{{{\rho}}_{\text{k}\left(\text{m}\right)\text{i}}-{{\rho}}_{\text{k}\left(\text{m}-1\right)\text{i}}}{{{\rho}}_{\text{k}\left(\text{m}-1\right)\text{i}}}$$

Where,

$$\:{{\pi}}_{\text{k}\left(\text{m}\right)\text{i}}=\text{I}\text{n}\text{f}\text{l}\text{a}\text{t}\text{i}\text{o}\text{n}$$

And,

$$\:{{\rho\:}}_{\text{k}\left(\text{m}\right)\text{i}}={{\rho\:}}_{\text{w}\left(\text{m}\right)\text{i}}+{{\rho\:}}_{\text{o}\left(\text{m}\right)\text{i}}+{{\rho\:}}_{\text{r}\left(\text{m}\right)\text{i}}+{{\rho\:}}_{\text{l}\left(\text{m}\right)\text{i}}$$

$$\:{{\rho\:}}_{\text{k}\left(\text{m}\right)\text{i}}=\text{T}\text{o}\text{t}\text{a}\text{l}\:\text{P}\text{r}\text{i}\text{c}\text{e}\:\text{o}\text{f}\:\text{C}\text{o}\text{m}\text{m}\text{o}\text{d}\text{i}\text{t}\text{i}\text{e}\text{s}\:\text{o}\text{f}\:\text{t}\text{i}\text{m}\text{e}\:\text{m}\:\text{a}\text{n}\text{d}\:\text{i}\text{t}\text{h}\:\text{d}\text{i}\text{v}\text{i}\text{s}\text{i}\text{o}\text{n}$$

$$\:{{\rho\:}}_{\text{w}\left(\text{m}\right)\text{i}}=\text{P}\text{r}\text{i}\text{c}\text{e}\:\text{o}\text{f}\:\text{W}\text{h}\text{e}\text{a}\text{t}\:\text{o}\text{f}\:\text{t}\text{i}\text{m}\text{e}\:\text{m}\:\text{a}\text{n}\text{d}\:\text{i}\text{t}\text{h}\:\text{d}\text{i}\text{v}\text{i}\text{s}\text{i}\text{o}\text{n}$$

$$\:{{\rho\:}}_{\text{o}\left(\text{m}\right)\text{i}}=\text{P}\text{r}\text{i}\text{c}\text{e}\:\text{o}\text{f}\:\text{O}\text{i}\text{l}\left(\text{P}\text{a}\text{l}\text{m}\right)\:\text{o}\text{f}\:\text{t}\text{i}\text{m}\text{e}\:\text{m}\:\text{a}\text{n}\text{d}\:\text{i}\text{t}\text{h}\:\text{d}\text{i}\text{v}\text{i}\text{s}\text{i}\text{o}\text{n}$$

$$\:{{\rho\:}}_{\text{r}\left(\text{m}\right)\text{i}}=\text{P}\text{r}\text{i}\text{c}\text{e}\:\text{o}\text{f}\:\text{R}\text{i}\text{c}\text{e}\left(\text{C}\text{o}\text{a}\text{r}\text{s}\text{e}\right)\:\text{o}\text{f}\:\text{t}\text{i}\text{m}\text{e}\:\text{m}\:\text{a}\text{n}\text{d}\:\text{i}\text{t}\text{h}\:\text{d}\text{i}\text{v}\text{i}\text{s}\text{i}\text{o}\text{n}$$

$$\:{{\rho\:}}_{\text{l}\left(\text{m}\right)\text{i}}=\text{P}\text{r}\text{i}\text{c}\text{e}\:\text{o}\text{f}\:\text{L}\text{e}\text{n}\text{t}\text{i}\text{l}\text{s}\left(\text{M}\text{a}\text{s}\text{u}\text{r}\right)\:\text{o}\text{f}\:\text{t}\text{i}\text{m}\text{e}\:\text{m}\:\text{a}\text{n}\text{d}\:\text{i}\text{t}\text{h}\:\text{d}\text{i}\text{v}\text{i}\text{s}\text{i}\text{o}\text{n}$$

Here, *i* = 1, 2, 3, …, 6; k = Types of commodities; m = Time (month) (1,2, …, 9).

For this analysis, we used a cross-sectional study. Our response variable is Haz, which is a continuous variable collected from the BDHS dataset. To determine whether inflation, as an explanatory variable, has any significant individual contribution to stunting, we use the following basic linear regression model:

$${\text{y}}_{\text{i}}={{\beta}}_{0}+{{\beta}}_{1}{\text{x}}_{\text{i}}+{{\varepsilon}}_{\text{i}}$$

$$\:{\text{y}}_{\text{i}}=\text{S}\text{t}\text{u}\text{n}\text{t}\text{i}\text{n}\text{g}\:\text{o}\text{f}\:\text{t}\text{h}\text{e}\:\text{C}\text{h}\text{i}\text{l}\text{d}\:\left(Haz\right)$$

$$\:{\text{x}}_{\text{i}}=\text{A}\text{v}\text{e}\text{r}\text{a}\text{g}\text{e}\:\text{I}\text{n}\text{f}\text{l}\text{a}\text{t}\text{i}\text{o}\text{n}\:$$

Furthermore, we run a multiple linear regression model with several additional explanatory factors such as child features, mother characteristics, and household characteristics.

$${\text{y}}_{\text{i}}={{\beta}}_{0}+{{\beta}}_{1}{\text{x}}_{\text{i}}+\sum\limits_{\text{i}=2}^{4}{{\beta}}_{\text{i}}{{\tau}}_{\text{i}}+\sum\limits_{\text{i}=5}^{12}{{\beta}}_{\text{i}}{{\upsilon}}_{\text{i}}+\sum\limits_{\text{i}=13}^{20}{{\beta\:}}_{\text{i}}{{\phi\:}}_{\text{i}}+{{\varepsilon}}_{\text{i}}$$

$$\:{\text{y}}_{\text{i}}=\text{S}\text{t}\text{u}\text{n}\text{t}\text{i}\text{n}\text{g}\:\text{o}\text{f}\:\text{t}\text{h}\text{e}\:\text{C}\text{h}\text{i}\text{l}\text{d}$$

$$\:{\text{x}}_{\text{i}}=\text{A}\text{v}\text{e}\text{r}\text{a}\text{g}\text{e}\:\text{I}\text{n}\text{f}\text{l}\text{a}\text{t}\text{i}\text{o}\text{n}\:$$

$$\:{{\tau}}_{\text{i}}=\text{C}\text{h}\text{i}\text{l}\text{d}\:\text{C}\text{h}\text{a}\text{r}\text{a}\text{c}\text{t}\text{e}\text{r}\text{i}\text{s}\text{t}\text{i}\text{c}\text{s}$$

$$\:{{\upsilon\:}}_{\text{i}}=\text{M}\text{o}\text{t}\text{h}\text{e}\text{r}\:\text{C}\text{h}\text{a}\text{r}\text{a}\text{c}\text{t}\text{e}\text{r}\text{i}\text{s}\text{t}\text{i}\text{c}\text{s}$$

$$\:{{\phi\:}}_{\text{i}}=\text{C}\text{h}\text{i}\text{l}\text{d}\:\text{C}\text{h}\text{a}\text{r}\text{a}\text{c}\text{t}\text{e}\text{r}\text{i}\text{s}\text{t}\text{i}\text{c}\text{s}$$

Finally, using the backward selection model procedure, we pick the following explanatory variables: wealth index, child gender, child height and weight, mother’s education level, whether the respondent is currently pregnant or not, and whether the respondent is currently breastfeeding or not. Following that, we run some model diagnostics on our model.

The fitted vs. residual plot demonstrates that the data are not normally distributed, and the Breusch-Pagan test reveals that our data is heteroscedastic. It could mean that one of our variables contains an outlier. To handle this situation, we eliminate outliers from the data using the formula:

$$\:{\text{z}}_{\text{i}}=\frac{{\text{x}}_{\text{i}}-\bar{\text{x}}}{{\sigma\:}}$$

About 1.2% of observations are removed by excluding outliers using the condition $\:\left|\text{z}\right|>3$. Nonetheless, because the residuals do not meet the homoscedasticity and linearity condition, we use a generalized linear regression model for the gaussian family in this research.

## Results

### Trends of inflation and stunting

**Fig. 2:**
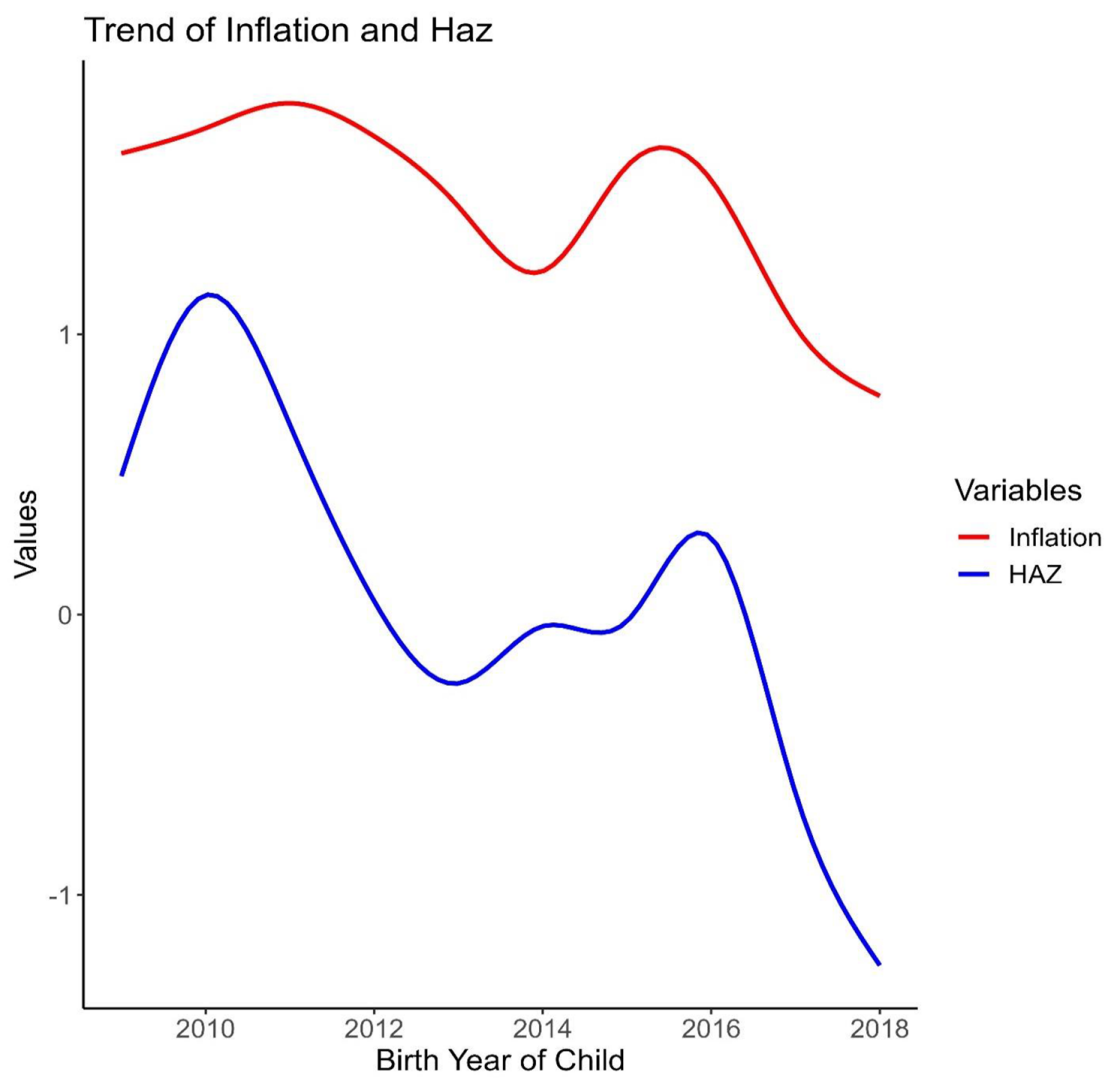
Trend of Inflation and HAZ throughout the years 2009 to 2018

**Fig. 3:**
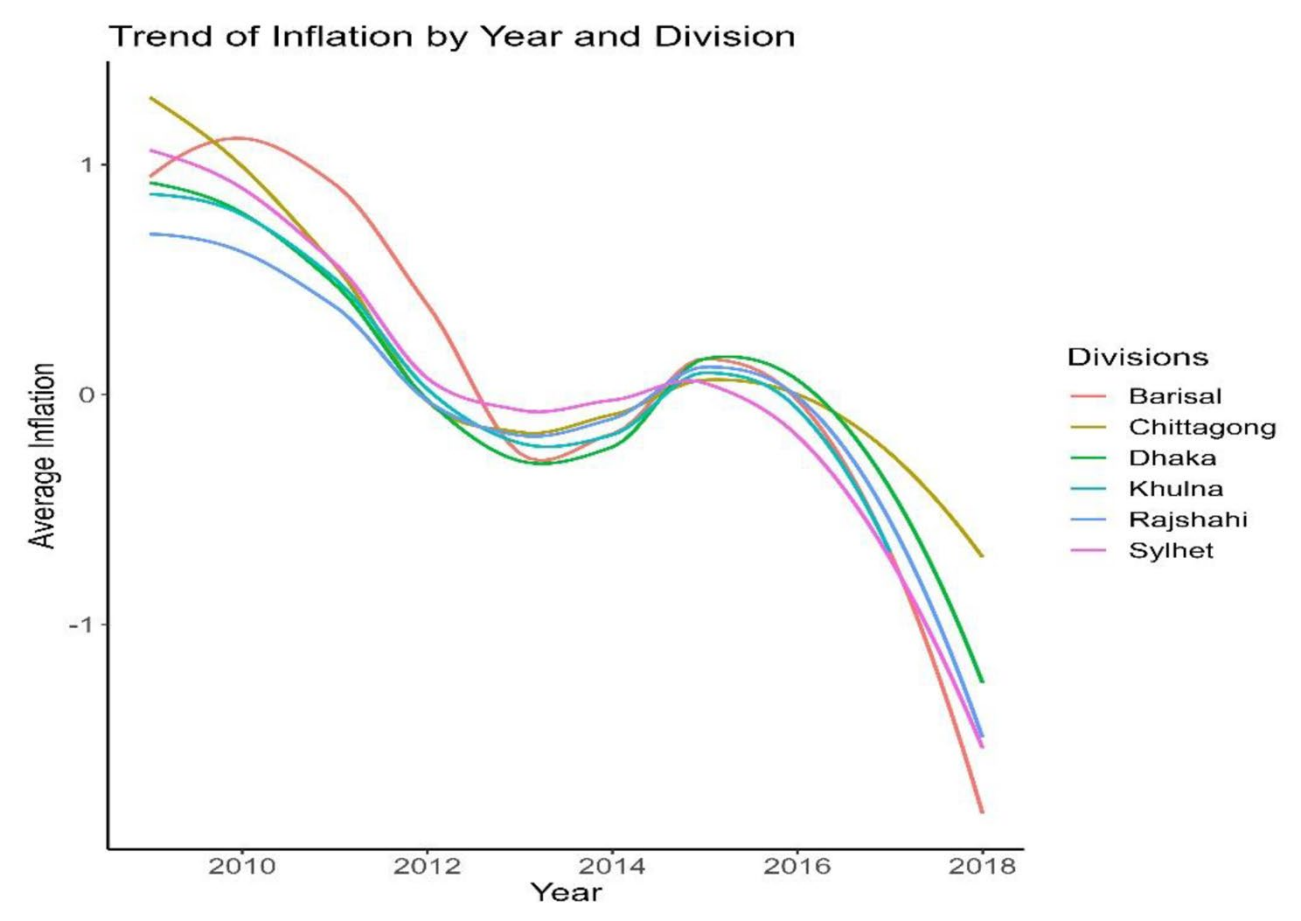
Trend of Inflation by year (2009–2018) and divisions

**Fig. 4:**
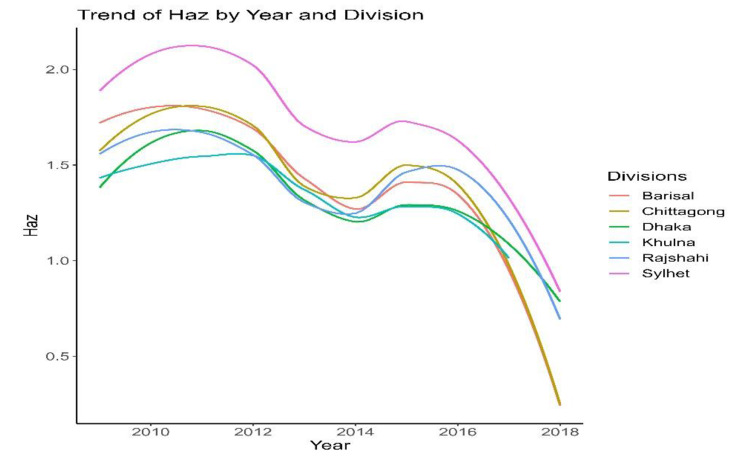
Trend of HAZ by year (2009–2018) and divisions

Line graph from Fig. 2 shows the decreasing trends of both inflation and stunting through the years 2009 to 2018. In 2009, the average food inflation was 0.48, and the average stunting for children under the age of five was − 1.67. The commodity’s average inflation was 1.10, and the average stunting was − 1.75 in 2010. However, stunting increased in 2011, when inflation was 0.63%. In 2012, there is 4% inflation, which is lower than the 2011. In the meantime, stunting was also decreasing. Average stunting in 2013 and 2014 was 1.46 and 1.19, respectively, whereas the difference between average inflation for 2013 and 2014 was 0.29. Stunting changed from − 1.19 (2014) to -1.62 in 2015 with a 0.02 change in inflation. Average Haz reduced in 2017 from the average Haz of 2016, with an inflation change of -0.88, that means both inflation and Haz decreased. A similar trend is also noticed in 2018 for stunting and inflation. The graph illustrates certain vicissitude tendencies, therefore it’s possible that there’s an association between them that needs further investigation to be confirmed. Also, it is evident from Fig. 3 and Fig. 4 that the regional variation in trends is similar to the overall trend.

### Descriptive analysis

Table 1 shows that (32.8%) of male children are stunted, compared to (33.0%) of female children, who are slightly more likely to be stunted than male children. Malnutrition is heavily influenced by one’s place of residence. The majority of stunted children (35.1%) reside in rural areas, while (27.5%) do so in urban areas. In particular, the Khulna division had the lowest percentage of stunted children in this study (25.9%), whereas the Sylhet division has the highest percentage (46.3%) of stunted children. Additionally, it was found that (29.9%) of children in Dhaka were stunted. Another crucial component in this study is the mother’s education level. The lowest percentage among mothers’ educational levels is found in children whose mothers hold a higher degree, with a stunting percentage of (17.1%). Stunted children are more likely to be born to uneducated mothers, with (45%) being the highest number in this category. The fact that the mother is currently pregnant is an essential component in this study. According to the data, (45.6%) of children are stunted if their mothers are currently pregnant, while (32.3%) are stunted if their mothers are not. Furthermore, (31.8%) of children whose mothers are now breastfeeding are stunted, while (34.4%) of children whose mothers are not breast feeding are stunted. HAZ is a continuous variable that indicates whether the children are stunting or not. If the result is less than − 2, the child is deemed stunted. According to Table 2 the minimum HAZ value is -5.40 and the greatest HAZ value is 2.45. The mean value of this variable is -1.49. Inflation is a significant variable in this study, with the lowest value of -1.57 and a mean value of 0.102. Height and weight are also key variables in this research. The children’s average height is 29.42 cm, and their average weight is 10.68 pounds.

**Table 1:** Frequency table of the characteristics

Variable	Stunting Status			
	Non-stunted		Stunted	
	Count	Row *N* %	Count	Row *N* %
Division				
Barisal	470	63.6	269	36.4
Chittagong	1812	65.5	956	34.5
Dhaka	2861	70.1	1222	29.9
Khulna	869	74.1	304	25.9
Rajshahi	1033	69.5	454	30.5
Sylhet	666	53.7	574	46.3
Total	7710	67.1	3780	32.9
Sex of Child				
Female	3702	67.0	1820	33.0
Male	4009	67.2	1960	32.8
Total	7710	67.1	3780	32.9
Mother’s Education Level				
higher	1061	82.9	219	17.1
no education	799	55.0	653	45.0
primary	1990	59.7	1344	40.3
secondary	3861	71.2	1564	28.8
Total	7710	67.1	3780	32.9
Mother’s pregnancy Status				
no or unsure	7420	67.7	3536	32.3
yes	290	54.4	244	45.6
Total	7710	67.1	3780	32.9
Currently breastfeeding				
no	3188	65.6	1669	34.4
yes	4522	68.2	2111	31.8
Total	7710	67.1	3780	32.9
Type of place of residence				
rural	5321	64.9	2875	35.1
urban	2389	72.5	905	27.5
Total	7710	67.1	3780	32.9

**Table 2:** Descriptive statistics of child characteristics and inflation

Variables	*N*	Minimum	Maximum	Mean	Std. Deviation
Haz	11,490	-5.40	2.45	-1.49	1.22
Waz	11,490	-5.51	4.50	-1.34	1.09
Whz	11,490	-5.00	4.99	− 0.71	1.13
Inflation	11,490	-1.57	1.91	0.10	0.55
Age of child	11,490	0	59	29.42	16.93
Weight of child	11,490	1.90	20.10	10.68	3.04

### Analysis (inflation)

In our first simple linear regression model, inflation has a significant effect on stunting, with a coefficient of -0.31 (95% CI: -0.36, -0.27) (*p* < 0.001) according to Table 3. That means, for every one unit increase in inflation HAZ decreases on average 0.31 units, resulting in child stunting. According to Table 6, the coefficient of determinant in our model is 0.02.

**Table 3:** Model-1 with inflation only

Coefficients	Estimate	Std. Error	t value	*p*-value
(Intercept)	-1.47(95% CI: -1.49, − 1.44)	0.01	-123.55	<0.001
Inflation	-0.31 (95% CI: -0.36, -0.27)	0.02	-15.15	< 0.001

### Analysis (with all variables)

Based on Table 4 our multiple linear regression model contains both significant and insignificant explanatory factors after integrating additional explanatory variables including child characteristics, mother characteristics, and household characteristics with AIC 7.74. A backward model selection process is used for variable selection, which trims down variables from a maximal model with AIC − 36.08. The maximal model here denotes a multiple linear regression model, assuming that the independent model contains errors. Finally, we chose the wealth index, the child’s gender, height and weight, the mother’s education level, whether the respondent is currently pregnant or not, and whether the respondent is currently breastfeeding or not.

**Table 4:** StepAIC procedure for variable selection for optimal model

Steps	AIC	Inflation	WI	Household head Sex	Shared Toilet	Sex	Weight	Age	Type of Residence	MEL	Drinking Water Source	Type of Toilet	Religion	Cooking Fuel Type	Daughters At Home	Sons Away from Home	M Preg	M B. Feed
1	7.74	✔	✔	✔	✔	✔	✔	✔	✔	✔	✔	✔	✔	✔	✔	✔	✔	✔
2	-9.91	✔	✔	✔	✔	✔	✔	✔	✔	✔	✖	✔	✔	✔	✔	✔	✔	✔
3	-18.43	✔	✔	✔	✔	✔	✔	✔	✔	✔	✖	✔	✔	✖	✔	✔	✔	✔
4	-23.09	✔	✔	✔	✔	✔	✔	✔	✔	✔	✖	✖	✔	✖	✔	✔	✔	✔
5	-27.22	✔	✔	✔	✔	✔	✔	✔	✔	✔	✖	✖	✖	✖	✔	✔	✔	✔
6	-29.21	✔	✔	✔	✔	✔	✔	✔	✔	✔	✖	✖	✖	✖	✔	✖	✔	✔
7	-31.12	✔	✔	✔	✖	✔	✔	✔	✔	✔	✖	✖	✖	✖	✔	✖	✔	✔
8	-32.99	✔	✔	✖	✖	✔	✔	✔	✔	✔	✖	✖	✖	✖	✔	✖	✔	✔
9	-34.64	✔	✔	✖	✖	✔	✔	✔	✖	✔	✖	✖	✖	✖	✔	✖	✔	✔
10	-36.08	✔	✔	✖	✖	✔	✔	✔	✖	✔	✖	✖	✖	✖	✖	✖	✔	✔

The final model output is reported in Table 5. According to the model, inflation has a significant effect on HAZ, with a coefficient of -0.13 (95% CI: -0.16, -0.09), suggesting that for every unit change in inflation, HAZ drops by 0.13 unit on average. The wealth index has a significant effect on stunting, according to this table. A poor-class family’s HAZ is 0.23 (95% CI: -0.3, -0.17) times lower than that of the richest group. A person from a lower, middle, or upper-class family has a HAZ that is on average 0.15 (95% CI: -0.22, -0.09), 0.11 (95% CI: -0.18, 0.05), and 0.10 (95% CI: -0.17, -0.05) units lower than someone from the richest category. Compared to female children, male children have a HAZ that is, on average, 0.22 (95% CI: -0.26, -0.19) units lower. The regression coefficient for the age variable is -0.07 (95% CI: -0.08, -0.07), indicating that there is a substantial increase in stunting with increasing child age. It is notable that HAZ increases by an average of 0.40 (95% CI: 0.39, 0.42) units for every unit increase in weight, indicating a lesser proclivity to malnutrition. The study shows that malnutrition decreases as the level of education increases. Those whose mothers are illiterate, have completed primary school, and have completed secondary school have a lower HAZ (0.28 (95% CI: -0.36, -0.2), 0.23 (95% CI: -0.3, -0.16), and 0.13 (95% CI: -0.19, -0.07) units) than those whose mothers are highly educated. Additionally, it is thought that children with pregnant mothers have a 0.23 (95% CI: -0.32, -0.14)-unit lower HAZ than children without pregnant mothers. The HAZ for children whose mothers are currently breastfeeding is 0.15 (95% CI: -0.21, -0.11) unit lower than for children whose mothers are not. The Gaussian family’s dispersion parameter is reported to be 0.99.

**Table 5:** Model-2 with additional variables

Coefficients	Estimate	Std. Error	t value	*p*-value
(Intercept)	-3.09 (95% CI: -3.22, -2.97)	0.06	-49.58	< 0.001
Inflation	-0.13 (95% CI: -0.16, -0.09)	0.02	-6.69	< 0.001
WI middle	-0.11 (95% CI: -0.18, 0.05)	0.03	-3.60	< 0.001
WI poorer	-0.15 (95% CI: -0.22, -0.09)	0.03	-4.60	< 0.001
WI poorest	-0.23 (95% CI: -0.30, -0.17)	0.03	-6.91	< 0.001
WI richer	-0.11 (95% CI: -0.17, -0.05)	0.03	-3.56	< 0.001
Sex (male)	-0.22 (95% CI: -0.26, 0.19)	0.02	-11.57	< 0.001
Age	-0.07 (95% CI: -0.08, -0.07)	0.00	-58.92	< 0.001
Weight	0.40 (95% CI: 0.39, 0.42)	0.01	63.09	< 0.001
MEL no education	-0.28 (95% CI: -0.36, -0.20)	0.04	-6.59	< 0.001
MEL primary	-0.23 (95% CI: -0.3, -0.16)	0.04	-6.51	< 0.001
MEL secondary	-0.13 (95% CI: -0.19, -0.07)	0.03	-4.08	0.03
M Preg (yes)	-0.23 (95% CI: -0.32, -0.14)	0.05	-5.06	< 0.001
M B.Feed (yes)	-0.16 (95% CI: -0.21, -0.11)	0.03	-6.13	< 0.001

**Table 6:** Model diagnostics outcomes

Model 1						
Residual Standard Error	Df	Multiple R-squared	Adjusted R-squared	F-statistic	Df	
1.22	10,913	0.02	0.02	229.60	1 and 10,913	

Model 2						
Dispersion Parameter for gaussian family	Null Deviance	Df	Residual Deviance	Df	AIC	
0.99	16,572	10,914	10,851	10,901	30,941	

Number of Fisher Scoring iteration: 2

**Fig. 5:**
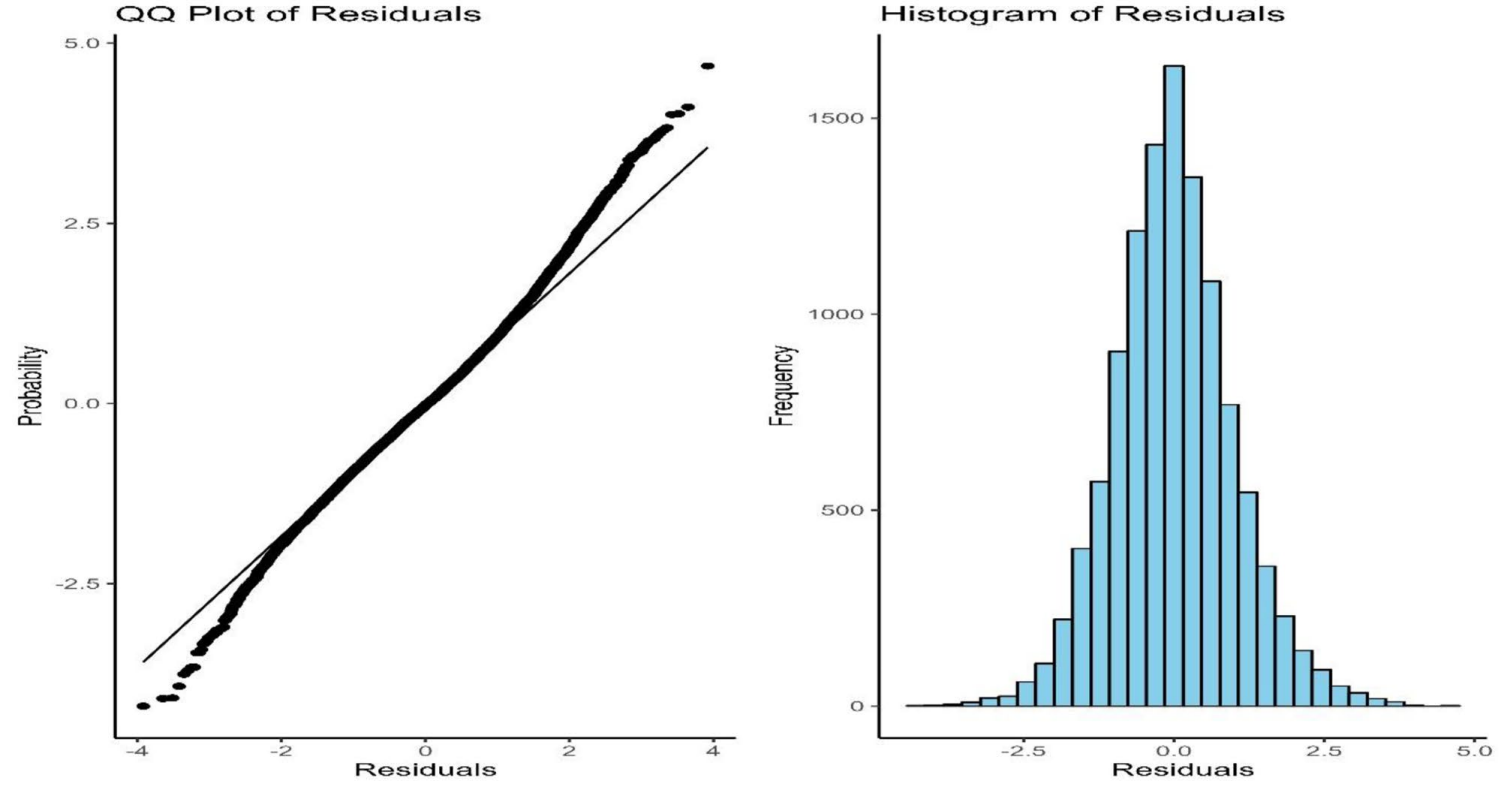
Model diagnosis using Q-Q plot and histogram of residuals

The primary goal of this study is to ascertain how inflation affects malnutrition. Two BDHS data sets combined with a price dataset are utilized for examination. The trendline graph shows an overall decreasing trend in both inflation and HAZ over the years. The graph indicates some ups and downs between 2010 and 2018. It is clear from that inflation may have a big impact on malnutrition. That is why a simple linear regression model was performed. According to this model, inflation has a significant impact on stunting, which is on the rise as a result of rising inflation. Since inflation is not the only factor influencing stunting, this model explains only a portion of its impact. As a result, we updated our model with a few new features. Only inflation is not enough to explain stunting fully. There are several other factors that affect stunting. Following that, we ran some diagnostics on our model (Table 6 and Fig. 5) to validate the assumption of multiple linear regression models. Figure 5 shows that residuals are randomly distributed to the horizontal axis, but a pattern line is also visible in this graph, which may indicate a non-linear relationship between them. The Brush-Pagan test indicates that the data does not meet the homoscedasticity assumption. The data is seen to be normally distributed by looking at the histogram graph and standard Q-Q plot (Fig. 5). Since our data exhibit heteroscedasticity, it is possible that some outliers contributed to the heteroscedasticity. To determine if there are any outliers, we then compute Cook distances. We run the same model after removing outliers, but the Brush-Pagan test still shows that heteroscedasticity is present. Finally, we run a generalized linear regression model for the Gaussian family with a dispersion parameter of 0.99, which is nearly 1 and is interpreted as unity variance.

## Discussion

In an Ethiopian study, Woldemichael et al. found a significant effect of food inflation on malnutrition. According to their study, for every 1% increase in food price inflation, the risk of stunting in children under five rises by 0.95%. In another study of them, the result shows that children between the ages of 6 and 59 months suffer negative medium- and long-term effects from being impacted by rising food prices [32, 33]. We also found a similar result in our study. Stunting drops by 0.13 units on average when food price inflation is increased by one unit.

In our analysis, inflation mostly refers to food inflation. Food inflation is the rising price of goods. Nonetheless, despite growing food prices, people’s real income has remained stagnant. People’s food purchase habits are affected as a result. Due to a lack of nutrients in the diet, the mother may experience malnutrition during pregnancy, which can cause malnutrition in the unborn child. Mothers suffer from numerous ailments because of a lower standard of living and are unable to seek medical care during pregnancy on time, which can harm the infant.

According to the GLM model’s findings, the wealth index has a significant effect on malnutrition. Malnutrition is more prevalent among poor children than among rich children because impoverished families may be unable to provide sufficient meals for their children or take good health care of them. Islam et al. studied the risk factor for childhood malnutrition. According to their findings, the wealth quantile has a significant impact on child malnutrition [26]. We find that malnutrition in children is more prevalent in females than in males. In Bangladesh, there is still gender disparity. The general consensus is that a boy’s nutritional needs are greater than a girl’s [34]. Age also has an impact on the prevalence of child malnutrition. The innate protection that a child receives from their mother’s breast milk diminishes as they become older owing to new eating habits. As a result, the child will experience illnesses like diarrhea, which eventually cause malnutrition [35]. Educated mothers are more conscious than illiterate mothers. They are more aware of new diseases or conditions as they are more updated by reading a newspaper or following a social media site. They can easily identify any critical situation and contact a doctor or other health worker. It makes it easier for educated mothers to take care of their children. As a result, the malnutrition of their children is less than that of those whose mothers are illiterate [36]. According to our research, children of uneducated mothers are more likely to be malnourished than children of primary, secondary, or higher-educated mothers. Pregnant or breastfeeding mothers may be unable to properly care for their children, making the children more vulnerable to malnutrition than others.

Since food price inflation has a significant effect on child malnutrition, the government can take a policy to mitigate the price in food. They also can take a policy for newborn child and pregnant women to ensure their healthy food. Investigating in health and nutrition programs for child and taking proper care of them can reduce the risk of suffering malnutrition.

### Limitations

Certain limitations to this study should be highlighted. Firstly, the study takes only price data from six major cities of Bangladesh, data for Rangpur was not accessible in the price dataset, and Mymensingh was part of the Dhaka division in the BDHS 2014. Moreover, data for all 64 districts are not totally available, thus we assumed the price of a product for a district based on its divisional price. Local market pricing can sometimes differ from town market prices. Additionally, road conditions, transportation facilities, and distance from the nearest market and healthcare facility are ignored because of data unavailability. Furthermore, each family’s daily nutritional intake was ignored, and it is uncertain whether a mother receives any rations specifically for her children. Also, some families are self-sufficient in food production, and inflation may have little or no impact on malnutrition in those households. This case is not included in the study. Lastly, a localized natural disaster simply affects localized health issues and price increases. Therefore, a comprehensive evaluation for that period cannot be finished without taking that into account.

However, the study was primarily concerned with determining whether inflation has a major impact on child malnutrition, which is rightly noted here. Children’s health could be compromised by inflation. This study shows the state of the economy and child health, which may lead to more in-depth research on the subject.

## Conclusion

We found that inflation has a significant negative effect on malnutrition in our analysis. Moreover, trend analysis shows the decreasing rate for stunting of the child under five aged children. The reason for which can be mainly attributed to the decrease in food inflation rate, increase in the education rate of women, increase in awareness in childbirth and childcare, ensuring health care for pregnant mothers etc. Even if inflation rises, the total income of household does not rise by the same amount. They then begin compromising in various ways such as cutting the cost of food, cloths, etc. to cover the additional expenditure. One of the consequences of this is child malnutrition. Our investigation attempted to bring this issue to light. Considering the issue of inflation is therefore important.

While immediate cost savings are not attainable, it is necessary to consider the cost of basic food items such as infant food and regular food that cannot be skipped. Furthermore, arrangements can be made for low-cost delivery of specific food to those who have children under the age of five at home. It is possible to educate illiterate parents on the nutritional importance of food and diet, as well as which foods meet a child’s nutritional needs on a limited budget. Above all, caution must be used to make sure that inflation is not detrimental to the child’s health.

### Electronic supplementary material

Below is the link to the electronic supplementary material.

Supplementary Material 1

## Data Availability

The Bangladesh Demographic and Health Survey (BDHS) data used in this study is publicly available on the Demographic and Health Surveys Program (DHS) website (https://dhsprogram.com). Also, the World Food Programme (WFP) data used in this study is publicly available on the WFP data website (https://data.humdata.org/m/dataset/wfp-food-prices-for-bangladesh).
