# Supplementary material for: Impact of food price inflation on stunting in under five aged children in Bangladesh
Source: Health Econ Rev. 2024 Aug 29;14:68. doi: 10.1186/s13561-024-00549-9 (PMC11363589; doi:10.1186/s13561-024-00549-9)
Supplement: Supplementary file 1 — Supplementary Material 1 [file 13561_2024_549_MOESM1_ESM.docx]

**Variables Detailed Information:**

| Variable | Description |
| --- | --- |
| Gender | Male  Female |
| Division | Barisal  Chittagong  Dhaka  Khulna  Rajshahi  Sylhet |
| Mother’s Education Level | Higher  No Education  Primary  Secondary |
| Mother’s pregnancy Status | No Or Unsure  Yes |
| Currently breastfeeding | No  Yes |
| Type of place of residence | Rural  Urban |
| Respondent's occupation as collected in the country | Agricultural Worker  Businessman  Farmer  Fisherman  Land Owner  Others |
| most recent partner or husband’s highest education level | Higher  No Education  Primary  Secondary  Others |
| Religion | Islam  Hinduism  Buddhism  Christianity  Others |
| Wealth Index | Richer  Richest  Middle  Poorer  Poorest |
| Toilet facilities shared with other households | Pit Latrine  Ventilated Improved Pit Latrine  Flush |
| Sex of the head of the household | Male  Female |
| Primary source of drinkable water by household members | Tubewell  Tap Water  River/Dam/Lake/Ponds/Stream/Canal/Irrigation Channel  Others |
| Toilet facility type in the household | Pit Latrine  Ventilated Improved Pit Latrine  Flush |
| Type of cooking fuel | Wood  Agricultural Crop  Natural Gas  LPG  Animal Dung  Others |
